# 

**Published:** 1916-09

**Authors:** 


*Good Morning, Doctor:—*

Want to do a "little deed of kindness" today?

Well, sit down and write out a check for your subscription to *Texas Medical Journal* and be sure you make it up to 1917—or 1920 if you wish. We have a number of subscribers who are paid up to 1920, and a larger number who do not wait to receive bills.

You will receive a receipt by return mail and the editor will be spared the time and expense of sending your bill. Every cent that comes in now is being put back in the Journal to make it better and better for you, so please hurry your subscription along today.

*EDITOR*

